# Laparoscopic-assisted distal colon excision and proximal colon pull-through anorectoplasty for anorectal malformation

**DOI:** 10.3389/fped.2024.1402666

**Published:** 2024-05-10

**Authors:** Siqi Li, Shiru Ye, Yan Zhou, Mei Diao, Long Li

**Affiliations:** ^1^ Department of Pediatric Surgery, Children’s Hospital Capital Institute of Pediatrics, Chinese Academy of Medical Sciences & Peking Union Medical College, Research Unit of Minimally Invasive Pediatric Surgery on Diagnosis and Treatment, Chinese Academy of Medical Sciences 2021RU015, Beijing, China; ^2^ Department of Pediatric Surgery, Tsinghua University Affiliated Beijing Tsinghua Changgung Hospital, Beijing, China

**Keywords:** anorectal malformation, laparoscopy, anorectoplasty, proximal colon pull-through, distal colon excision

## Abstract

**Purpose:**

During the second stage surgery for anorectal malformations (ARM), patients whose distal intestine of the colostomy is particularly short underwent laparoscopic-assisted distal colon excision and proximal colon pull-through anorectoplasty (PCPARP). This study aimed to discuss the outcomes of PCPARP after colostomy in patients with ARM.

**Methods:**

This is a single-center propensity score-matched (PSM) study which was retrospectively initiated patients with intermediate- or high-type ARM who underwent laparoscopic surgery from June 2007 to December 2018. These patients were divided into PCPARP group and conventional laparoscopic-assisted anorectoplasty (LAARP) group according to specific surgical methods. The general data, surgical data, postoperative complications, and functional results were evaluated.

**Results:**

In total, 216 patients were included in this study: 190 (88.0%) undergoing LAARP approach and 26 (12.0%) undergoing PCPARP approach. After PSM, two well-balanced groups of 26 patients were analyzed and showed the postoperative complications (*P* = 0.126) and bowel function (*P* = 0.809) were similiar between the two groups.

**Conclusions:**

The curative effect of PCPARP after colostomy is similar to that of classic LAARP surgery, which can be used for ARM patients with a very short and abnormal distal intestine of the stoma.

## Introduction

Congenital anorectal malformations (ARM) are common congenital anomalies occurring in 1 in 5,000 births and have a spectrum of anatomical presentations, requiring individualized treatments for the newborn, and sophisticated approaches to the definitive reconstruction (1). The introduction of laparoscopic-assisted anorectoplasty (LAARP) for high-type ARM was first reported in 2000 by Georgeson et al. (2). Subsequent studies (3–5) have confirmed the safety and efficacy of LAARP. In 2020, our center published the experience with LAARP in 330 cases of ARM over a 20-year period, highlighting its benefits in rectal mobility and recovery, intra-regional fibrous closure, and accurate tunnel formation in the long-term muscle tube with minimal trauma, and improved patient prognosis in intermediate and high type ARM cases (6).

During laparoscopic surgery, some patients may exhibit anomalies in the distal intestines of the colostomy, such as extreme shortness or morphological abnormalities like pouch colon, abnormal thickening, abnormal dilation, and severe adhesions. Such intestines may have peristalsis dysfunction, and may increases the risk of constipation (7–9). In such cases, complete excision of the colon and rectum at the distal end of the stoma was applied, followed by pulling the colon out through the center of the sphincter for anorectoplasty at the proximal stoma end. We term this surgical approach laparoscopic-assisted distal colon excision and proximal colon pull-through anorectoplasty (PCPARP). However, concerns have been raised regarding the potential increase in rectal prolapse incidence due to extensive perirectal dissection (10). Therefore, further research is needed on the safety and prognosis of PCPARP.

In this study, we aimed to discuss the outcomes of the PCPARP for intermediate- and high-type ARM, comparing it with traditional LAARP.

## Materials and methods

### Patients

**Figure 1:**
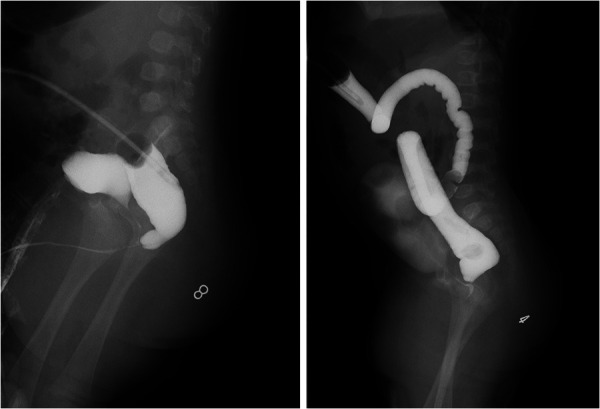
The high-pressure distal colostogram before anorectoplasty.

Ethics approval from the Ethics Board of the Capital Institute of Pediatrics (China) was obtained. Written consent was obtained from the patients' parents before surgery. The medical records of patients with intermediate- or high-type ARM who underwent laparoscopic surgery from June 2007 to December 2018 in our center were retrospectively analyzed. The associated defects, wingspread classification, age at the operation, weight at the operation, colostomy situation, operative time, length of hospital stay, length of postoperative hospital stay, length of bowel resection, time of colostomy closure, as well as postoperative complications (including wound infection, recurrent fistula, urethral diverticulum, anal stenosis and rectal prolapse, etc.), were recorded. All patients underwent the high-pressure distal colostogram before surgery to determine the location of the fistula and the morphology of the distal colon (Figure 1).

### Surgical procedures

All these patients underwent colostomy surgery after birth at local hospitals.

The patient was positioned supine for laparoscopic access. One trocar (5-mm) was inserted in the umbilicus and two 3-mm trocars were inserted 3–4 cm away from the left and the right of the first port. Dissection of the rectum began laterally at about 1–2 cm proximal to the peritoneal reflection using a monopolar hook cautery. As the fistula approached, the dissection was made in the submucosa layer of the rectal pouch. The anterior layer of the terminal rectum was opened and the orifice of the fistula was identified from the direction of rectum mucosal folds. The mucosal layer of the rectum was transected at the junction. Then closing the fistula by suturing the muscular cuff with a running suture of 5-0 PDS. Determine the next surgical plan based on the length, width, thickness, and blood supply of the distal intestine at the stoma.

#### LAARP

The patient's legs were elevated to the lithotomy position. Using the electrical simulator to determine the center position of the sphincter and make a vertical incision. Then a curved Kelly clamp was used to make the tunnel. The rectum was grasped by the clamp and gently pulled through the tunnel down to the perineum. The rectum was pulled out as much as possible, and the redundant and/or abnormal rectum was trimmed. The anastomosis between the rectum and anus was completed with circumferential interrupted 5-0 PDS sutures.

#### PCPARP

**Figure 2:**
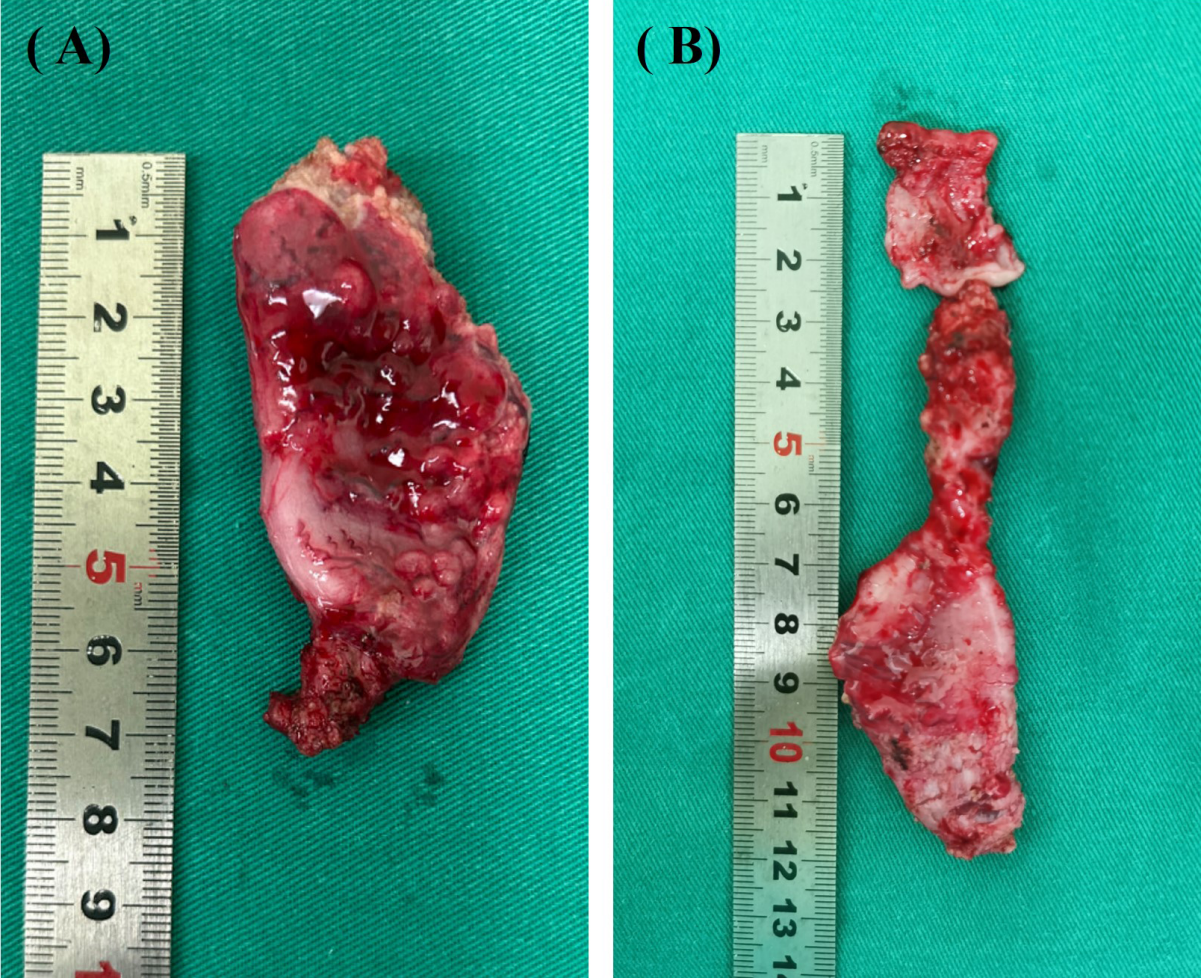
Gross specimen of the intestine at the distal end of the stoma. (**A**) Severe thickening and dilation of the intestine. (**B**) Pouch colon.

PCPARP is selected due to the following conditions: (1) Less distal intestinal, (2) severe thickening or dilation of the rectum (Figure 2A), (3) pouch colon (Figure 2B), (4) severe intestinal adhesion, (5) poor blood supply to the distal intestine, causing the distal intestine of the stoma is too short to be pulled down to the perineum. The next step was to form the tunnel from the perineal aspect similar to LAARP, and leave the clamp in the tunnel. Enter the abdominal cavity from the surgical incision of the stoma, free the distal colon and remove it, and free the proximal colon to the appropriate length. The proximal colon was grasped by the clamp and gently pulled through the tunnel down to the perineum, and then sutured it with the anus.

### Outcome assessment

Due to the unique nature of the medical environment, we regularly follow up over the phone, recording patients' bowel movements and providing guidance. To evaluate functional outcomes, we used the Krickenbeck classification (11) and the Bowel Function Score (BFS) questionnaire (12). The Krickenbeck classification includes three parameters: voluntary bowel movements, soiling, and constipation. Voluntary bowel movements were defined as feeling the urge to defecate, the capacity to verbalize this feeling, and the ability to hold the bowel movement. Soiling was graded as follows: Grade 1, occasional soiling (up to once or twice per week); Grade 2, soiling every day without social problems; and Grade 3, constant soiling with social problems. Constipation was graded as follows: Grade 1, constipation manageable by changes in diet; Grade 2, constipation requiring laxatives; and Grade 3, constipation resistant to laxatives and diet.

### Statistical analysis

Statistical analysis was performed by SPSS 26.0. Continuous variables were presented as the mean with standard deviation or median and interquartile range (IQR). Categorical variables were reported as counts and percentages. Pearson's χ^2^ test, Fisher's exact test, two independent samples *t*-tests and the non-parametric Mann-Whitney *U*-test were used to compare characteristics between two groups. Propensity score matching (PSM) was used to minimize selection bias. The patients were matched using relevant variables (the classification, sacral deformity, tethered cord, and stoma site) to of equate the complexity of the surgical cases. A matched group of patients was created with a 1:1 ratio. The PSM method is closest to the neighborhood method having a caliper width of 0.20. *P *< 0.05 was considered statistically significant.

## Results

During a 10-year study period, a total of 216 patients were included, with 190 (88.0%) undergoing LAARP and 26 (12.0%) undergoing PCPARP. Among them, there were 100 (41.2%) cases with high-type ARM and 116 (47.7%) cases with intermediate-type ARM. The median age at the operation was 3.9 (3.4, 5.0) months with an average weight of 6.6 ± 1.2 kg. Patients in the LAARP group underwent stoma reversion of colon surgery at 3.1 ± 0.5 months postoperatively. The baseline characteristics of LAARP vs. PCPARP before and after PSM analysis are summarized in Table 1. Before the PSM analysis, all the patients were analyzed, and the proportion of sigmoidostomy in the PCPARP group was significantly higher than that in the LAARP group (76.9% vs. 40.0%, *P *< 0.001).

**Table 1:** Baseline characteristics before and after propensity score matching analysis.

Baseline characteristics	All patients			Propensity-matched patients		
	PCPARP	LAARP	*P*-value	PCPARP	LAARP	*P*-value
	*N* = 26	*N* = 190		*N* = 26	*N* = 26	
Age at anorectoplasty (months)	3.6 (3.2, 5.4)	3.9 (3.4, 5.1)	0.414	3.6 (3.2, 5.4)	3.4 (2.9, 4.2)	0.164
Body weight at anorectoplasty (kg)	7.0 ± 1.0	6.5 ± 1.2	0.056	7.0 ± 1.0	6.5 ± 1.2	0.086
Age at follow-up (years)	8.0 (4.5, 11.9)	6.3 (4.6, 7.7)	0.106	8.0 (4.5, 11.9)	7.4 (6.3, 9.3)	0.874
ARM classification						
Intermediate-type	12 (46.2)	104 (54.7)	0.410	12 (46.2)	12 (46.2)	1.000
High type	14 (53.8)	86 (45.3)		14 (53.8)	14 (53.8)	
Type of fistula						
Rectovesical fistula	7 (26.9)	21 (11.1)	0.065	7 (26.9)	5 (19.2)	0.478
Rectoprostatic fistula	6 (23.1)	64 (33.7)		6 (23.1)	9 (34.6)	
Rectobulbar fistula	11 (42.3)	99 (52.1)		11 (42.3)	13 (46.2)	
No fistula	2 (7.7)	6 (3.2)		2 (7.7)	0	
Associated defects						
Cardiovascular	2 (7.7)	10 (5.3)	0.612	2 (7.7)	0	0.490
Urogenital	10 (38.5)	68 (35.8)	0.790	10 (38.5)	11 (42.3)	1.000
Sacral deformity	2 (7.7)	24 (12.6)	0.468	2 (7.7)	4 (15.4)	0.668
Tethered cord	0	8 (4.2)	0.600	0	0	–
Sacral ratio	0.68 (0.66, 0.75)	0.71 (0.65, 0.76)	0.895	0.68 (0.66, 0.75)	0.71 (0.69, 0.77)	0.399
Colostomy						
Transverse colostomy	6 (23.1)	114 (60.0)	**<0** **.** **001**	6 (23.1)	6 (23.1)	1.000
Sigmoid colostomy	20 (76.9)	76 (40.0)		20 (76.9)	20 (76.9)	

Bold values are statistically significant.

LAARP, laparoscopic-assisted anorectoplasty; PCPARP, proximal colon pull-through anorectoplasty.

After PSM, two well-balanced groups of 26 patients were analyzed. Operative findings and postoperative complications after a PSM analysis of LAARP vs. PCPARP are reported in Table 2. The PCPARP group had significant longer operative time (*P *= 0.013), length of hospital stay (*P *< 0.001), and length of intestinal resection (*P *= 0.001) compared to the LAARP group. The median postoperative follow-up time was 8.0 (4.5, 11.9) and 7.4 (6.3, 9.3) years. There was no significant difference in the incidence of postoperative complications between the two groups (38.5% vs. 19.2%, *P* = 0.126).

**Table 2:** Operative findings and postoperative complications after propensity score matching analysis.

	PCPARP	LAARP	*P*-value
	*N* = 26	*N* = 26	
Surgical time (h)	3.0 ± 0.7	2.4 ± 0.7	**0** **.** **013**
Hospitalization time (days)	20 (16, 24)	15 (11, 16)	**<0** **.** **001**
Postoperative hospitalization time (days)	11 (8, 13)	7 (6, 8)	**<0** **.** **001**
Resected intestinal length (cm)	6.5 (3.0, 9.5)	3.0 (2.0, 4.3)	**0** **.** **001**
Postoperative complications	10 (38.5)	5 (19.2)	0.126
Rectal prolapse	7 (26.9)	4 (15.4)	0.308
Wound infection	1 (3.8)	0	1.000
Intestinal obstruction	0	1 (3.8)	1.000
Urethral diverticulum	1 (3.8)	0	1.000
Anal stenosis	0	0	–
Recurrence of fistula	0	0	–
Perianal infection	1 (3.8)	0	1.000

Bold values are statistically significant.

LAARP, laparoscopic-assisted anorectoplasty; PCPARP, proximal colon pull-through anorectoplasty.

A total of 36 patients were evaluated for bowel function, including 17 (65.4%) in the PCPARP group and 19 (73.1%) in the LAARP group. The postoperative bowel function evaluation results of these patients are shown in Table 3. The mean BFS results were comparable between the two groups (15.29 ± 2.14 vs. 15.11 ± 2.47, *P* = 0.809). No differences in voluntary bowel movements (*P* = 0.650), soiling (*P* = 0.957) and constipation (*P* = 0.450) were observed between two groups. Specifically, voluntary bowel movements were present in 14 (82.3%) PCPARP and 17 (89.5%) LAARP patients. Nine (53.0%) PCPARP and 12 (63.2%) LAARP patients did not have soiling or Grade I soiling. The incidence of no constipation in PCPARP group is higher than that in LAARP group, even if the results were not statistically significant (64.7% vs. 36.8%). Similarly, the PCPARP group had a lower incidence of Grade I (5.9% vs. 15.8%), Grade II (23.5 vs. 36.8%), and Grade Ⅲ constipation (5.9% vs. 10.5%) compared to the LAARP group. In addition, only one patient in the PCPARP group reported diagnosed neurogenic bladder, while the remaining patients did not report urinary dysfunction.

**Table 3:** Postoperative bowel function results for two groups.

		PCPARP	LAARP	*P*-value
		*N* = 17	*N* = 19	
The BFS scores		15.29 ± 2.14	15.11 ± 2.47	0.809
The Krickenbeck classification				
Voluntary bowel movements		14 (82.3)	17 (89.5)	0.650
Soiling	No	2 (11.8)	2 (10.5)	0.957
	Grade 1	7 (41.2)	10 (52.6)	
	Grade 2	3 (17.6)	3 (15.8)	
	Grade 3	5 (29.4)	4 (21.1)	
Constipation	No	11 (64.7)	7 (36.8)	0.450
	Grade 1	1 (5.9)	3 (15.8)	
	Grade 2	4 (23.5)	7 (36.8)	
	Grade 3	1 (5.9)	2 (10.5)	

LAARP, laparoscopic-assisted anorectoplasty; PCPARP, proximal colon pull-through anorectoplasty.

## Discussion

The conventional treatment plan for intermediate- and high-type ARM is mostly the three-stage approach, a temporary sigmoid or transverse colostomy is initially created followed by anoplasty in several months, and then the colostomy is closed several weeks to months later (13). At present, LAARP is widely used in intermediate- or high-type ARM, especially in cases with a high-position fistula and poor neuromuscular development (rectovesical fistula, rectoprostatic fistula, and rectovaginal fistula). Its main advantages are minimal muscle damage and minimal incision infection (14).

Due to China's vast territory and unique medical environment, most ARM patients undergo emergency colostomy at local hospitals before being referred to specialized centers for anorectoplasty. High pressure distal colostogram was performed routinely before anorectoplasty to assess the condition of the distal colon and fistula. As mentioned in methodology, improper colostomy in the neonatal period, severe postoperative intra-abdominal adhesions, or abnormal morphology of the intestinal tract itself make us have to abandon the distal end of the colostomy site of these patients.

The PSM analysis can be used in observational/retrospective cohort studies reducing selection bias by equating the groups compared. Thus, it allows reliable results and robust evidence, avoiding randomized controlled trials [18]. By applying the PSM analysis, the study demonstrated that the PCPARP group had significantly higher surgical time and postoperative hospitalization time than the LAARP group, which is understandable given the need for additional surgical steps and complexity in cases selected for PCPARP. Closure of a colostomy is often performed at local hospitals, and we are unable to obtain hospitalization data for statistical analysis. In 2019, a two-stage LAARP was reported, and the results showed that the total surgical time, total postoperative hospitalization time, and total hospital expenses were all lower than those of the three-stage LAARP [19].

Notably, the incidence of postoperative complications did not significantly differ between the two groups, underscoring the safety of PCPARP despite its technical intricacies. The incidence of rectal prolapse was comparable between two groups (26.9% vs. 15.4%, *P* = 0.308), both lower than previous literature reports (30%–59%) (15, 16). In previous studies comparing PSARP and LAARP, it is believed that extensive perirectal dissection over the longer length of the rectum and non-fixation of the distal rectum to the muscle complex in LAARP may be the reason for a higher incidence of rectal prolapse (10). According to the experience of our center, maintaining the correct tension of rectum-anal anastomosis while placing the anoplasty sutures could reduce the rate of rectal prolapse. Ming et al. (17) reported the incidence of rectal prolapse 1 year after LAARP was significantly decreased in the modified group (3.7%) compared with the conventional group (35.3%) for ARM. This may be the reason why the incidence of rectal prolapse did not significantly increase in the PCPARP group.

The main goals in the management of ARM patients are to achieve fecal continence and prevent constipation. Regarding functional outcomes, both PCPARP and LAARP demonstrated comparable results in terms of voluntary bowel movements, soiling, and constipation. At present, there is still controversy over whether resection of the distal rectum can improve intestinal function in children. Some scholars believe that the blind end of the rectum in children with ARM has a bowel storage function and an internal sphincter-like structure. Removing it may increase the risk of soiling (18, 19), but our results do not show such a trend. However, histopathological studies of the distal rectum have found that preserving the intestine which has inherent neuromuscular defects increases the risk of constipation (7–9). Gangopadhyay et al. observed the presence of disrupted muscularis mucosa (10%), thickened muscularis mucosa (3.3%), and striated muscles (10%) in the distal rectal pouches, and these may be implicated in the pathogenesis of postoperative constipation (8). There are also studies indicating that ganglia absence in the terminal rectum of the male imperforate anus is associated with constipation after anoplasty (7, 20). A study in 2013 showed that the severe anomalies of the muscularis propria along with the abnormal microscopic organization of the connective tissue are consistent with the high rate of constipation (21). Our center found that preserving the distal end of the rectum is a risk factor for constipation in ARM patients, possibly related to residual intestinal fibrosis, by comparing the prognosis of two groups of patients with intestinal resection greater than and less than 3 cm (Accepted but unpublished results). This study showed that the incidence of constipation in the PCPARP group was lower than that in the LAARP group (35.3% vs. 63.2%), which may be due to the complete resection of the intestinal tissue with structural abnormalities. However, further research is needed to determine whether the distal end of the rectum should be removed and what abnormal distal rectum cannot be preserved.

The present study has some limitations: the retrospective design, the small sample size, and the small number of PCPARP. PSM analysis was applied to mitigate selection bias, thus avoiding randomization and assuring reliable results. Nonetheless, our study underscores the importance of individualized surgical approaches in managing ARM and highlights PCPARP as a valuable adjunct to traditional LAARP in select cases. It is imperative to exercise caution in interpreting these findings, as PCPARP should be considered a specialized technique rather than a replacement for LAARP. Although there is no increase in the morbidity rate of postoperative complications and the functional outcomes are equivalent to those of LAARP. At the same time, we believe that more caution should be exercised in the treatment of colostomy surgery for ARM patients during their neonatal period. Avoid inappropriate colostomy, avoid intestinal adhesions, and thoroughly clean fetal feces in the distal intestinal tract, thereby reducing the impact on the second stage surgery.

## Data Availability

The raw data supporting the conclusions of this article will be made available by the authors, without undue reservation.
